# Host-Fungal Interactions: Pathogenicity versus Immunity

**DOI:** 10.1155/2012/562480

**Published:** 2012-05-27

**Authors:** Arianna Tavanti, Julian R. Naglik, Nir Osherov

**Affiliations:** ^1^Department of Biology, University of Pisa, Via San Zeno 37, 56127 Pisa, Italy; ^2^Department of Oral Medicine, Pathology, and Immunology, King's College London Dental Institute, King's College London, London SE1 9RT, UK; ^3^Department of Clinical Microbiology and Immunology, Sackler School of Medicine, Tel-Aviv University, Ramat-Aviv, Tel-Aviv 69978, Israel

Fungal pathogens represent a leading cause of life-threatening infections in debilitated patients and exert a substantial toll on health care resources. A major development in the past few years, resulting from extremely rapid technological advances, has been the elucidation of the genome sequences of all of the major human fungal pathogens. Together with the development of transcriptomics and proteomics, fungal pathogen research has entered the so-called “omics” era. However, despite the remarkable acceleration in the understanding of many molecular mechanisms underlying phenomena such as drug resistance, fungal virulence, and pathogenesis, many key aspects of the host-fungal interactions are still not fully understood.

This current issue provides in-depth review articles drafted by international experts tackling different perspectives of the host-pathogen interplay. Their contributions offer insights into aspects related to fungal virulence and drug resistance of both established and emerging fungal pathogens, as well as models to study their pathogenicity.

The paper by G. P. Moran and colleagues explains why *Candida albicans* has evolved to become such a successful opportunistic pathogen, by comparative global gene expression analysis performed on *C. albicans* and its most closely related species *C. dubliniensis*.

The epidemiology of invasive fungal infections has evolved over the past twenty years. Yeasts other than *C. albicans* and moulds other than *Aspergillus fumigatus* have emerged as significant causes of invasive mycoses in severely immunocompromised patients. Tools allowing a prompt diagnosis for these emerging pathogenic fungi are lacking, often leading to a delay in effective treatment and high mortality rates. This is the case for invasive mucormycosis, a rapidly progressing infection often refractory to antifungal therapy, with a negative prognosis in individuals with impaired immune defence. The key role of iron uptake, angioinvasion, and neurotropism in the virulence repertoire of this fascinating group of fungi is reviewed by G. Morace and E. Borghi.

Chitin is one of the most abundant biopolymers in nature and comprises part of the fungal cell wall structure. Although humans do not biosynthesize chitin, they do express chitin degrading enzymes. The targeted recognition of fungal chitin by the host may have implications for diagnostic assays, as well as for potential new therapeutic approaches. This emerging topic is reviewed by K. Vega and M. Kalkum. Another major process that influences fungal cell wall structure is protein glycosylation, which is essential for eukaryotic cells. However, the mechanisms by which carbohydrates play a role in the development of fungal diseases are still poorly characterised. In this context, C. Jin provides an overview on the contribution of protein glycosylation to growth, cell wall synthesis and development in *A. fumigatus*, possibly providing a novel strategy for drug development.

*Histoplasma capsulatum* is a major fungal pathogen endemic to the Americas, exhibiting a broad variety of clinical presentations, ranging from mild lung infection to life-threatening systemic infections. The severity of infection and its outcome result from a complex interaction between the virulence repertoire of the pathogen and the host's immune defences. The paper by M. R. Mihu and J. D. Nosanchuk focuses on the dynamic host-pathogen interplay in the context of the virulence arsenal displayed by the fungus and the innate and adaptive immune responses of the host.

The molecular mechanisms underlying the ability of dermatophytes to establish infection and persist in the host are reviewed by R. R. Achterman and T. C. White. Despite the fact that this group of filamentous fungi are the most common cause of cutaneous mycoses, very little is known about dermatophyte pathogenesis. The paper presents the current research status on dermatophyte virulence factors.

The ability of fungal cells to chemically communicate with each other and with other microorganisms has only recently been discovered. The paper by F. Cottier and F. A. Mühlschlegel depicts a fascinating picture of a closely connected microbial community in the environment, where multispecies communication takes place. Versatile fungal communicative competences are thought to have a significant impact on several fungal biological functions such as mating, growth, or the regulation of virulence factor expression. Among the latter, hyphal formation has been widely recognised to significantly contribute to the pathogenesis of several fungal species, by promoting adhesion to biotic or abiotic surfaces and tissue penetration. The paper by A. Brand presents the current knowledge on *C. albicans* hyphal growth during infection, addressing the issue of specific functions and mechanical/structural properties conferred by hyphal formation during infection.

 Fungal pathogens have evolved complex mechanisms of resistance to antifungal drugs. The nature, frequency, and molecular mechanisms of resistance to currently used antifungals are the focus of the review by P. Vandeputte and colleagues, who also describe the latest approaches to develop new antifungal strategies.

A growing number of fungal pathogens have been associated with life-threatening infections resulting from biofilm-related infections that are particularly difficult to treat. Indeed, biofilm-embedded fungal cells are far more resistant to antifungal agents than their corresponding planktonic cells. The molecular basis of this remarkable resistance to antifungals has yet to be fully elucidated. In this respect, G. Ramage and coworkers review the latest findings on this topic and shed new light on the multifactorial nature of biofilm antifungal resistance. From a different perspective, H. Tournu and P. Van Dijck describe some of the most recent approaches shown to eradicate *Candida* biofilms, based on novel strategies to evaluate biofilm formation in vitro and in vivo.

The choice of the most appropriate experimental infection model to use for fungal pathogenesis-, virulence-, immunology-, and therapy-based studies is critical. In this issue, several contributions were made towards this topic. Mucosal and systemic models of *Candida* infection and virulence are discussed in depth by D. M. MacCallum, while the range of experimental models currently available for cryptococcosis is reviewed by W. Sabiiti and coworkers. G. Hamilos and colleagues offer a critical analysis of the use of *Drosophila melanogaster* as a surrogate model to study the immunopathogenesis of fungal infection and illustrate the recent advances in the study of medically important filamentous fungi in *Drosophila*.

Bioluminescent imaging is a powerful tool to perform real time evaluation of temporal and spatial progression of infections. Mainly used for the study of bacterial infection process, M. Brock discusses the application of bioluminescent imaging to the study of mycoses, summarising the key features of different luciferase systems and providing suggestions for future applications.

We believe that the collection of reviews authored by experts in the field presented here will provide a timely update of recent rapid developments in the field of medical mycology. We hope that the reviews will be helpful in assembling the interlocking pieces of the complex jigsaw that is the fungal-host interplay, as well as provide a basis for the generation of novel ideas and hypotheses for future experimentation.

**Arianna Tavanti** **Arianna Tavanti** **Julian R. Naglik** **Julian R. Naglik** **Nir Osherov** **Nir Osherov**

